# Promoter CpG methylation in cancer cells contributes to the regulation of *MUC4*

**DOI:** 10.1038/sj.bjc.6604845

**Published:** 2009-01-06

**Authors:** N Yamada, Y Nishida, H Tsutsumida, M Goto, M Higashi, M Nomoto, S Yonezawa

**Affiliations:** 1 Department of Human Pathology, Field of Oncology, Kagoshima University Graduate School of Medical and Dental Sciences, 8-35-1 Sakuragaoka, Kagoshima 890-8544, Japan; 2 Department of Clinical Pathology, Kagoshima Medical Center, 8-1 Shiroyamatyou, Kagoshima 892-0853, Japan

**Keywords:** *MUC4*, mucin, epigenetics, DNA methylation, massARRAY

## Abstract

Mucin 4 (MUC4) is a high molecular weight transmembrane mucin that is overexpressed in many carcinomas and is a risk factor associated with a poor prognosis. In this study, we show that the DNA methylation pattern is intimately correlated with *MUC4* expression in breast, lung, pancreas and colon cancer cell lines. We mapped the DNA methylation status of 94 CpG sites from −3622 to +29 using MassARRAY analysis that utilises base-specific cleavage of nucleic acids. MUC4-negative cancer cell lines and those with low MUC4 expression (eg, A427) were highly methylated near the transcriptional start site, whereas MUC4-positive cell lines (eg, NCI-H292) had low methylation levels. Moreover, 5-aza-2′-deoxycytidine and trichostatin A treatment of MUC4-negative cells or those with low MUC4 expression caused elevation of *MUC4* mRNA. Our results suggest that DNA methylation in the 5′ flanking region play an important role in *MUC4* gene expression in carcinomas of various organs. An understanding of epigenetic changes in *MUC4* may contribute to the diagnosis of carcinogenic risk and prediction of outcome in patients with cancer.

Mucins are high molecular weight glycoproteins that have oligosaccharides attached through O-glycosidic linkages to serine or threonine residues of the core protein. Many human mucin core proteins (MUC1-MUC9, MUC11-13 and MUC15-20) have been identified, (Lapensee *et al*, 1997; Yonezawa and Sato, 1997; Gum *et al*, 2002; Pallesen *et al*, 2002; Yin *et al*, 2002) including MUC4, a large transmembrane mucin with a very long glycosylated extracellular domain that is expressed in various normal tissues (Audie *et al*, 1993, 1995; Buisine *et al*, 1999; Gipson *et al*, 1999). MUC4 is also often overexpressed in epithelial cancers and our immunohistochemical studies have shown that aberrant expression of MUC4 is associated with invasive proliferation of tumours and a poor outcome for patients (Shibahara *et al*, 2004; Saitou *et al*, 2005; Tamada *et al*, 2006; Tsutsumida *et al*, 2007).

*MUC4* also serves as a novel intramembrane ligand for the receptor tyrosine kinase ErbB2, a transmembrane glycoprotein with a tyrosine kinase domain that is encoded by the c-ErbB-2 proto-oncogene and is highly homologous with the epidermal growth factor receptor (Yamamoto *et al*, 1986). Furthermore, *MUC4* plays an important role in cell proliferation and differentiation of epithelial cells by inducing specific phosphorylation of ErbB2 and enhancing the expression of p27 (Jepson *et al*, 2002), a cyclin-dependent kinase inhibitor that regulates the G1 and S phases of the cell cycle (Polyak *et al*, 1994).

We recently described the epigenetic regulation of the *MUC1* gene (Yamada *et al*, 2008). Similarly to *MUC1*, MUC4 is a factor associated with poor prognosis in several carcinomas (Shibahara *et al*, 2004; Saitou *et al*, 2005; Tamada *et al*, 2006; Tsutsumida *et al*, 2007), and thus we hypothesised that *MUC4* is also regulated by an epigenetic mechanism. To investigate the possible epigenetic regulation of *MUC4* gene expression, we mapped the DNA methylation status of the *MUC4* promoter region using 10 cancer cell lines derived from carcinomas of four different organs (breast, lung, pancreas and colon). Methylation of cytosine in genomic DNA plays an important role in gene regulation, and especially in gene silencing (Bird, 1992), and generally the promoter region of a transcribed gene is hypomethylated (Wolffe *et al*, 1999; Stirzaker *et al*, 2004).

To examine the methylation profiles of 92 CpG sites in the *MUC4* promoter in the cancer cell lines, we performed a MassARRAY methylation analysis (Ehrich *et al*, 2005). On the basis of the results of this analysis, methylation-specific polymerase chain reaction (PCR; MSP) primers were designed to ensure that particular CpG sites were related to gene expression. *MUC4*-negative cells or those with low *MUC4* expression were also treated with a DNA methylation inhibitor, 5-aza-2′-deoxycytidine, and a histone deacetylase inhibitor, trichostatin A (TSA), to confirm that DNA methylation and histone modification suppressed the expression of *MUC4* mRNA. Using these results, we describe an epigenetic mechanism through which *MUC4* gene expression is tightly linked to DNA methylation in several organs.

## Materials and methods

### Cells and treatment

Human breast cancer cell lines MCF-7 (MUC4+/−), T-47D (MUC4+/−) and MDA-MB-453 (MUC4+/−); human lung cancer cell lines NCI-H292 (MUC4+) and A427 (MUC4-); human pancreatic carcinoma cell lines HPAFII (MUC4+), BxPC3 (MUC4+) and PANC1 (MUC4+/−) and human colon adenocarcinoma cell lines LS174T (MUC4+/−) and Caco2 (MUC4+/−) were obtained from American Type Culture Collection (Manassas, VA, USA). MCF-7, A427, HPAFII, Caco2 and LS174T cells were cultured in Eagle’s minimum essential medium (Sigma, St Louis, MO, USA); PANC1 cells were cultured in D-MEM (Sigma); T-47D, NCI-H292 and BxPC3 cells were cultured in RPMI-1640 medium (Sigma) and MDA-MB-453 cells were cultured in Leibovitz’s L-15 medium (Invitrogen, Carlsbad, CA, USA). All media were supplemented with 10% foetal bovine serum (Invitrogen) and 100 U ml^−1^ penicillin 100 *μ*g ml^−1^ streptomycin (Sigma). MUC4-negative cells or cells with low MUC4 expression were split 24 h before treatment. MDA-MB-453, PANC1 and LS174T cells were incubated with 100 *μ*M 5-aza-2′-deoxycytidine (5-AzadC; Sigma) and/or 500 nM TSA(Sigma) for 5 days, MCF-7, T-47D and Caco2 cells were incubated with 100 *μ*M 5-AzadC and/or 50 nM TSA for 5 days, and A427 cells were incubated with 1 *μ*M 5-AzadC and/or 50 nM TSA for 5 days. Media were changed every 24 h.

### Quantitative reverse transcription PCR (RT—PCR) analysis

Messenger RNA from cells that had or had not been treated with 5-AzadC, TSA or 5-AzadC/TSA in combination was purified with an RNeasy Mini kit (Qiagen, Valencia, CA, USA). Of a total of 100 *μ*l of mRNA, 20 *μ*l was reverse transcribed with Random-Hexamers (Applied Biosystems, Foster City, CA, USA). A 3.5-*μ*l cDNA aliquot was amplified in 25 *μ*l of 2xTaqMan universal master mix, 2.5 *μ*l of 20xTarget assay mix and 2.5 *μ*l of 20x control assay mix (Applied Biosystems) under the following PCR conditions: 2 min at 50°C, 10 min at 95°C, 45 cycles for 15 s at 95°C and 1 min at 60°C. The primers and probes were designed and synthesised by Applied Biosystems. The Target assay mix used for MUC4 had the product number Hs003666414. Human GAPDH (product number 4310884E) was used to calibrate the original concentration of mRNA; that is, the concentration of mRNA in the cell was defined as the ratio of target mRNA copies relative to *GAPDH* mRNA copies. In this analysis, data from three separate experiments were averaged.

### *MUC4* gene promoter sequencing

Genomic DNA was extracted from the 10 cell lines using a DNeasy tissue system (Qiagen) according to the manufacturer’s instructions. DNA was PCR amplified using seven pairs of sense and antisense primers (Table 1) for the full-length *MUC4* promoter. Polymerase chain reaction fragments were sequenced using a single-strand sequencing method (Hokkaido System Science Co., Hokkaido, Japan). Sequences were analysed with an ABI Prism 310 Genetic Analyzer (PE Applied Biosystems).

### Quantitative methylation analysis

Quantitative methylation analysis of the *MUC4* promoter was performed using the MassARRAY compact system (Hitachi high technologies corporation, Tokyo, Japan; Ehrich *et al*, 2005; Yamada *et al*, 2008). DNA from the cell lines was extracted using a DNeasy tissue system (Qiagen). A 1-mg sample of DNA was converted with sodium bisulfite using an EZ DNA methylation kit (Zymo research, Orange, CA, USA) and the modified DNA was amplified by PCR. The target regions were amplified using the primer pairs shown in Table 1. Each forward primer was tagged with a 10mer (5′-AGGAAGAGAG-3′) to balance the PCR, and each reverse primer had a T7-promoter tag (5′-CAGTAATACGACTCACTATAGGGAGAAGGCT-3′) for *in vitro* transcription. Polymerase chain reaction amplification was performed with the following parameters: hot start at 94°C for 15 min, followed by denaturing at 94°C for 20 s, annealing at 56°C for 30 s, extension at 72°C for 1 min for 45 cycles and final incubation at 72°C for 3 min. Unincorporated dNTPs were dephosphorylated by adding 2 *μ*l of premix including 0.3 U shrimp alkaline phosphate (SAP; Sequenom, San Diego, CA, USA). The reaction mixture was incubated at 37°C for 40 min and SAP was then heat inactivated for 5 min at 85°C. After SAP treatment, 2 *μ*l of the PCR products were used as a template for *in vitro* transcription, and RNase A cleavage was used for the reverse reaction, following the manufacturer's instructions (Sequenom). The samples were conditioned and spotted on a 384-pad Spectro-CHIP (Sequenom) using a MassARRAY nanodispenser (Samsung, Irvine, CA, USA), followed by spectral acquisition on a MassARRAY analyzer compact MALDI-TOF MS (Sequenom). The resultant methylation calls were analysed with EpiTyper software v1.0 (Sequenom) to generate quantitative results for each CpG site or an aggregate of multiple CpG sites.

### DNA extraction and DNA MSP analysis

DNA from the cell lines was extracted using a DNeasy tissue system (Qiagen) according to the manufacturer’s instructions. Bisulfite modification of the genomic DNA was carried out using a Epitect Bisulfite Kit (Qiagen), and the modified DNA was amplified by PCR using a AmpliTaq Gold Fast PCR Kit (Applied Biosystems). The target regions were amplified using the primer pairs shown in Table 1. The PCR conditions were 95°C for 10 min, 40 cycles at 96°C for 3 s, 59°C for 3 s and 68°C for 3 s, with a final extension reaction at 72°C for 10 s. The amplified products were subjected to 1.5% agarose gel electrophoresis.

## Results

### Effects of 5-AzadC and TSA on *MUC4* mRNA expression

**Figure 1:**
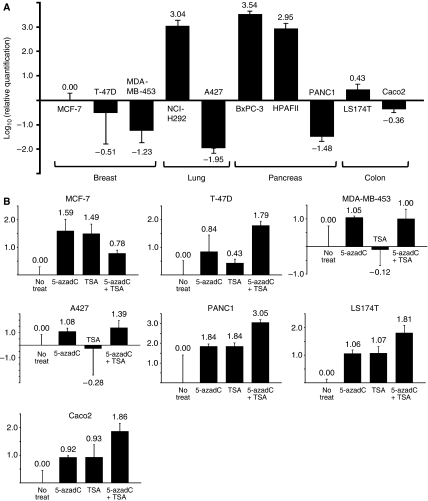
Quantitative RT–PCR analysis of 10 cancer cell lines before and after treatment. (**A**) Expression of *MUC4* mRNA examined by quantitative RT–PCR. The bar graphs show gene expression levels relative to those in MCF-7 cells. NCI-H292, BxPC-3 and HPAFII cells showed high expression of *MUC4* mRNA, whereas MCF-7, T-47D, MDA-MB-453, A427, PANC1, LS174T and Caco2 cells had no or low levels of *MUC4* mRNA. (**B**) Quantitative analysis of *MUC4* mRNA in cells with little or no *MUC4* expression after treatment with 5-AzadC, TSA and 5-AzadC/TSA in combination. The bar graphs show gene expression levels as log_10_ values relative to those in untreated cells. After 5-AzadC or TSA treatment, MCF-7 cells showed significant restoration of *MUC4* mRNA expression. In T-47D cells, 5-AzadC/TSA treatment was most effective in restoring expression. *MUC4* mRNA levels were markedly restored in MDA-MB-453 and A427 cells after treatment with 5-AzadC or 5-AzadC/TSA. In PANC1, LS174T and Caco2 cells, 5-AzadC/TSA treatment was more effective in restoring expression compared to treatment with 5-AzadC or TSA alone.

The expression levels of *MUC4* mRNA in the human breast cancer cell lines MCF-7, T-47D and MDA-MB-453, the human lung cancer cell lines NCI-H292 and A427, the human pancreatic carcinoma cell lines HPAFII, BxPC-3 and PANC1 and the human colon adenocarcinoma cell lines LS174T and Caco2 were examined using quantitative RT–PCR analysis (Figure 1A). NCI-H292, BxPC-3 and HPAFII cells expressed *MUC4* mRNA, but MCF-7, T-47D, MDA-MB-453, A427, PANC1, LS174T and Caco2 cells did not do so. To examine whether DNA methylation and histone modification suppress the *MUC4* mRNA expression level, MUC4-negative cells or cells with low expression of MUC4 were treated with a DNA demethylating agent, 5-AzadC, a histone deacetylase inhibitor, TSA or 5-AzadC/TSA in combination (Figure 1B). Quantitative RT–PCR analysis of MCF-7 breast cancer cells showed that treatment with 5-AzadC or TSA gave a greater increase in *MUC4* mRNA compared to treatment with 5-AzadC/TSA in combination (38.9-, 30.9- and 6.03-fold increases, respectively). In contrast, in T-47D cells, the combination treatment was more effective than 5-AzadC or TSA alone in increasing *MUC4* mRNA (61.7-, 6.92- and 2.69-fold increases, respectively). Following 5-AzadC or 5-AzadC/TSA treatment of MDA-MB-453 cells, *MUC4* mRNA expression increased by 11.2- and 10.0-fold, respectively, but TSA alone had no effect. Treatment of A427 cells with 5-AzadC or 5-AzadC/TSA resulted in an increase in *MUC4* mRNA of 12.0- to 24.5-fold, whereas the level remained constant after treatment with TSA alone. In PANC1, LS174T and Caco2 cells, treatment with 5-AzadC or TSA increased the *MUC4* mRNA levels and 5-AzadC/TSA in combination further increased these levels (by 69.2-, 69.2- and 1,120-fold, respectively, in PANC1 cells; 11.5-, 11.7- and 64.6-fold, respectively, in LS174T cells; and 8.32-, 8.51- and 72.4-fold, respectively, in Caco2 cells). These results suggest that DNA methylation and/or histone modification influence *MUC4* gene expression.

### DNA sequencing of the human *MUC4* mucin gene promoter

**Figure 2:**
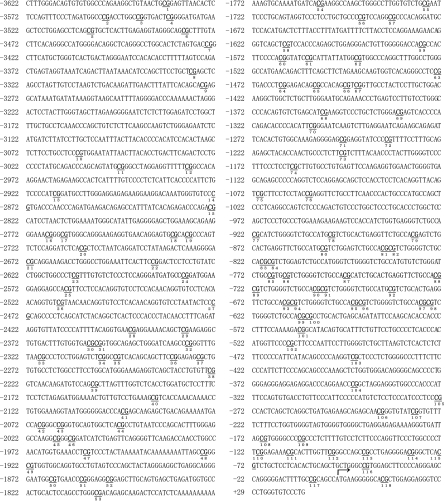
The human *MUC4* gene promoter sequence, which spans positions −3,622 to +29 with respect to the transcription start site. The numbers of CpG sites and the transcriptional start site +1 (arrow) are shown. CpG sites 53–76 were undetectable by MassARRAY analysis in the 10 cell lines. Between bases −833 to −648, sequence variation was observed among the 10 cell lines.

To investigate whether *MUC4* gene expression is regulated by SNPs, sequencing primers were designed to target different regions of the *MUC4* promoter (Table 1) based on the published sequence (Perrais *et al*, 2001). We sequenced a 3.6-kb promoter region of the human *MUC4* from 10 cell lines (Figure 2) and found that bases at some positions differed among the 10 cell lines. However, there was no correlation between these sequence differences and *MUC4* gene expression.

### Quantitative MassARRAY methylation analysis of the *MUC4* gene promoter in 10 cancer cell lines

**Figure 3:**
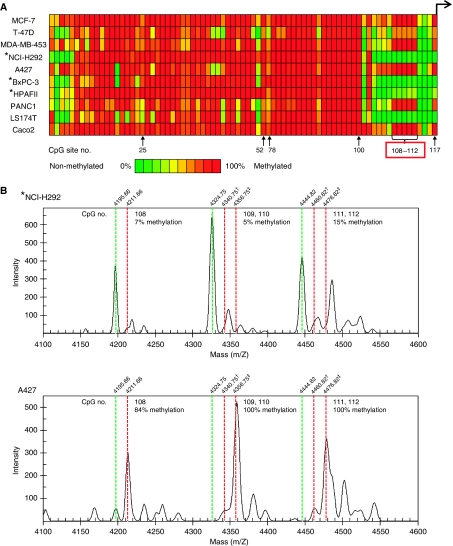
Summary of the CpG methylation status of the *MUC4* gene promoter region in 10 cancer cell lines. (**A**) Quantitative methylation analysis of CpG sites located in the *MUC4* promoter using a MassARRAY compact system. Different colours display relative methylation changes in 10% increments (green=0%, red=100% methylated). CpG sites that correlated well with *MUC4* expression are boxed in red. (**B**) Raw spectrum data for CpG sites 108–112 in lung cancer cell lines. Non-methylated and methylated CpG sites generate status-specific mass signals. Methylation status (%) is calculated as (intensity of methylated CpG (red dotted line)/(intensity of methylated CpG (red dotted line)+intensity of unmethylated CpG (green dotted line))). ^†^ Indicates methylation in one of the two CpG sites (eg, 109 or 110). ^‡^ Indicates methylation of both CpG sites (eg, 109 and 110). Asterisks indicate MUC4-positive cell lines.

Quantification of promoter DNA methylation levels was performed using the MassARRAY compact system, with mapping of the resulting data (Figure 3A). Samples were analysed by matrix-assisted laser desorption ionisation time-of-flight mass spectrometry (MALDI-TOF-MS), which permits high-throughput identification of methylation sites and semiquantitative measurement at single or multiple CpG sites. The raw spectrum data for CpG sites 108–112 in NCI-H292 and A427 lung cancer cells are shown in Figure 3B. In the vicinity of the transcriptional start site, MUC4-negative cells or cells with low expression of MUC4 showed high methylation (MCF-7, T-47D, MDA-MB-453, A427, PANC1 and Caco2) compared with MUC4-positive cell lines (NCI-H292, BxPC-3 and HPAFII). In contrast, the *MUC4* promoter CpG sites were mostly hypomethylated in LS174T cells, which showed low expression of MUC4. In the 10 cancer cell lines, methylation of CpG sites 108–112 was inversely correlated with *MUC4* gene expression, whereas methylation of sites 1–107 was almost unrelated to gene expression. These results indicate that the CpG methylation status near to the *MUC4* transcriptional start site may play an important role in gene expression of *MUC4*.

### Methylation-specific PCR analysis of *MUC4* promoter methylation in 10 cancer cell lines

**Figure 4:**
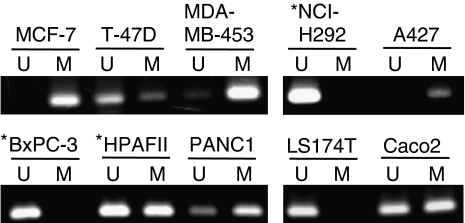
Methylation-specific PCR (MSP) analysis of the *MUC4* gene promoter region in 10 cancer cell lines. The PCR products labelled as ‘M’ (methylated) were generated by methylation-specific primers and those labelled as ‘U’ (unmethylated) were generated by primers specific for unmethylated DNA. The unmethylated allele was detected in MUC4-positive NCI-H292 and BxPC-3 cells, whereas the methylated allele was detected in MUC4-negative/low MCF-7, MDA-MB-453, A427 and PANC1 cells. Asterisks indicate MUC4-positive cell lines.

Methylation-specific PCR primers were designed to target CpG sites in the 5′ flanking region of the *MUC4* promoter (Table 1) based on the results of MassARRAY analysis. The five CpG sites (Nos. 108–112) included in the MSP primer showed 4–39% methylation in MUC4-positive cells and 63–100% methylation in all MUC4-negative/low cells, except for LS174T cells. The LS174T cells showed 5–19% methylation at the five sites. To confirm the reliability of the MSP primer, MSP analysis was performed on the 10 cell lines (Figure 4). An unmethylated band (lanes indicated by U in Figure 4) was obtained in MUC4-positive NCI-H292 and BxPC-3 cells, whereas a methylation band (lanes indicated by M in Figure 4) was observed in MUC4-negative/low MCF-7, MDA-MB-453, A427 and PANC1 cells. The MSP data were almost consistent with the results of MassARRAY analysis of the methylation status of the cell lines. Therefore, our results suggest that the 5′ flanking region of the promoter, and especially CpG sites 108–112, may play an important role in methylation-related gene silencing of *MUC4*.

## Discussion

We have previously examined the expression profiles of MUC4 in primary tumours of patients with intrahepatic cholangiocarcinoma-mass forming type (*n*=27; Shibahara *et al*, 2004), pancreatic adenocarcinoma (*n*=135) (Shibahara *et al*, 2004; Saitou *et al*, 2005; Tamada *et al*, 2006; Tsutsumida *et al*, 2007), extrahepatic bile duct carcinoma (*n*=70; Tamada *et al*, 2006) and small-sized lung adenocarcinoma (less than 3 cm; *n*=185; Tsutsumida *et al*, 2007) and compared MUC4 expression with the survival of the patients. In the four series, survival in MUC4-positive or high-expression cases was significantly worse than that for MUC4-negative or low-expression cases, and multivariate analyses showed that MUC4 expression in the carcinomas was a risk factor for a poor prognosis (Shibahara *et al*, 2004; Saitou *et al*, 2005; Tamada *et al*, 2006; Tsutsumida *et al*, 2007).

The regulatory mechanism of the *MUC4* gene is unclear. Initially, to examine the possible epigenetic regulation of *MUC4* expression, we treated MUC4-negative or low-expression cancer cell lines with 5-AzadC and/or TSA (Figure 1B). In four of seven cells (T-47D, PANC1, LS174T and Caco2), treatment with 5-AzadC/TSA significantly restored the *MUC4* mRNA expression, compared to the treatment with 5-AzadC or TSA alone. In MCF-7 cells, however, the restoration level of *MUC4* mRNA decreased in 5-AzadC/TSA treatments rather than in treatment with 5-AzadC or TSA alone. Yet, it is unclear whether 5-AzadC and TSA caused an antagonistic effect in MCF-7 cells. We showed that treatment with TSA did not restore the *MUC4* mRNA in MDA-MB-453 and A427 cells. Kondo *et al* (2003) showed that 5-AzadC or a combination of 5-AzadC and TSA, but not TSA alone, reactivates tumour suppressor gene expression at silenced loci (eg*, p16*). Our results in MDA-MB-453 and A427 cells are in good agreement with their observation. Although there were differences in the restoration level of *MUC4* mRNA among treated cell lines, our overall results suggested the possibility that *MUC4* expression is regulated by epigenetic mechanisms.

Next, to rule out the contribution of SNPs in control of *MUC4* expression, the sequences of the *MUC4* promoter were verified in all the cells used in the study using primers based on the published sequence (Perrais *et al*, 2001). In Figure 2, the sequence of part of the promoter (−833 to −647) could not be determined by single-strand sequencing, and we were also unable to obtain reliable results for one promoter region (−1822 to −973) using the MassARRAY compact system. Moreover, we found that bases at some positions differed among the 10 cell lines in a 3.6-kb *MUC4* promoter region. However, we found no correlation between these sequence differences and *MUC4* expression, and therefore we examined the DNA methylation status of the *MUC4* promoter in 10 cancer cell lines.

In this study, we used MassARRAY analysis to examine the methylation status of 92 CpG sites in the promoter of the *MUC4* gene. Methylation at CpG sites 108–112 (−170 to −102) was associated with the expression of *MUC4* in all cell lines except for LS174T. MUC4-negative cell lines were hypermethylated at these five CpG sites, whereas MUC4-positive cell lines showed hypomethylation at the same sites. We recently reported that *MUC1* expression in pancreatic, breast and colon cancer cells is regulated by DNA methylation and histone H3-K9 modification in the 5′ flanking region, also using a MassARRAY compact system. Methylation of CpG near the transcriptional start site (−100 to 100) was inversely correlated with *MUC1* gene expression (Yamada *et al*, 2008). Similarly, *MUC4* expression was also regulated by methylation of CpG sites near the transcriptional start site (−170 to −102).

On the other hand, the methylation status of upstream regions clearly differed between *MUC1* and *MUC4*. In *MUC1*, most CpG sites except for the regulatory region were unmethylated (Yamada *et al*, 2008); in contrast, in *MUC4,* almost all CpG sites of the upstream region (until CpG site 100) were methylated (Figure 3A). The reason for the different global methylation levels of these similar transmembrane mucins requires further investigation.

We were unable to show a relationship between DNA methylation status and expression of the *MUC4* gene in the LS174T colon cancer cell line, similarly to our results for *MUC1* (Yamada *et al*, 2008). However, expression of *MUC4* in Caco2 cells, which are derived from cancer cells of the same organ, was related to the DNA methylation status. This indicates that the regulation of expression by DNA methylation is not organ-specific, but varies in individual cell lines.

Recently, Vincent *et al* (2008) reported that *MUC4* expression is regulated epigenetically through DNA methylation and histone modification in pancreatic and gastric epithelial cancer cell lines, and that CpG sites 110–114 (−121 to −81) were associated with expression of *MUC4* in these cells (Vincent *et al*, 2008). However, in our study, CpG 113 (−93) and 114 (−81) were hypomethylated in all cell lines and unrelated to the expression of *MUC4* gene, whereas sites 108 and 109 were associated with *MUC4* gene expression. These differences might be due to different methods of detection, and it also possible that these CpG sites (108, 109, 113 and 114) may be sensitive and unstable. This issue may be resolved by the use of different analytical methods in future studies.

MUC4 is a factor associated with a poor prognosis in various carcinomas and particularly in intractable carcinomas, such as pancreatobiliary and lung carcinomas (Saitou *et al*, 2005; Tamada *et al*, 2006; Tsutsumida *et al*, 2007). This study shows that *MUC4* expression is regulated by DNA methylation in the promoter region, and we have also shown that MUC1, another prognostic indicator, is regulated by a similar epigenetic mechanism (Yamada *et al*, 2008). These results indicate that MassARRAY is a powerful tool for detection of methylation of CpG sites, and screening of the DNA methylation status of *MUC1* and *MUC4* genes by MassARRAY analysis of discharged fluids, such as pancreatic juice, bile or sputum is likely to be of value for early detection of pancreatobiliary and lung carcinomas.

## Figures and Tables

**Table 1:** Synthetic oligonucleotides used in the study

**Name**	**Primer sequence**	**Position**
*MUC4 promoter sequencing primers*		
MUC4-S1L	AGGCAGAACAGAGCTCAAATTC	−3647 to −3626
MUC4-S1R	ACAAAATGAGTGGCTTCTCTAGTTC	−2970 to −2946
MUC4-S2L	ACTTACACCTGACTTCAGACTCCTG	−3047 to −3023
MUC4-S2R	TTCCTCGTTCACCTGTAAAATG	−2,410 to −2389
MUC4-S3L	AACCAGGTGTCGTAACAACAAG	−2523 to −2504
MUC4-S3R	ACCATCTCAGCTCACTGCAAG	−1846 to −1826
MUC4-S4L	AAGAGCTGACAGAAAAATGACCAC	−2,092 to −2,069
MUC4-S4R	TGTAGCTCTCTGCAAAGGAAAAC	−1236 to −1214
MUC4-S5L	CCATGAACAGACTTTCAGCTTCTAG	−1521 to −1497
MUC4-S5R	CCAGATGGTGGACTTCTTCTTTC	−958 to −936
MUC4-S6L	CCTACCGAGGTTTTGCCTTC	−1063 to −1044
MUC4-S6R	CTAATCACCCTTTTCTCTCCTCAG	−194 to −171
MUC4-S7F	CATACAGTGCATTTCTGTTCCTG	−557 to −535
MUC4-S7R	ATCACTTACCTGGGACCACATG	+70 to +91
		
*10mer-tagged or T7-tagged primers* a		
MUC4-1L	aggaagagagTGTGTGGTTTAGAAGGTTGTAATTG	−3611 to −3587
MUC4-1R	cagtaatacgactcactatagggagaaggctACCCAAATAAAAATCCCTAATTTTT	−3233 to −3208
MUC4-2L	aggaagagagTGGGAGAATTTTATGATTTTATTTTTGTT	−3134 to −3106
MUC4-2R	cagtaatacgactcactatagggagaaggctATTTCCAAACCCAAATCTTTCCTAC	−2671 to −2647
MUC4-3L	aggaagagagGTAGGAAAGATTTGGGTTTGGAAAT	−2671 to −2647
MUC4-3R	cagtaatacgactcactatagggagaaggctAACATCCAAATAAAACCAAACTAAAC	−2204 to −2179
MUC4-4L	aggaagagagTGTGTGTTTTTGGTTTTTTTGGTAT	−2274 to −2250
MUC4-4R	cagtaatacgactcactatagggagaaggctACACCATCTCAACTCACTACAAACTC	−1849 to −1824
MUC4-5L	aggaagagagTAGTTTTTTGTTTTGGAAAGAAGAAGTTTA	−973 to −944
MUC4-5R	cagtaatacgactcactatagggagaaggctAACAAACCCCAAAAAATTAAAAAAC	−512 to −488
MUC4-6L	aggaagagagGGTTTGTTATGTGTTTGGGATTTG	−793 to −770
MUC4-6R	cagtaatacgactcactatagggagaaggctAAAATAAATAAACCACCCTCCTAAC	−344 to −320
MUC4-7L^b^	aggaagagagTAGGGATATTTAGGGGATTTTTTTT	+18 to +42
MUC4-7R^b^	cagtaatacgactcactatagggagaaggctTTCCCAACAACCCAAAACTCTAATA	−415 to −391
		
*MSP primers*		
MSP-UL^c^	GGTGATTAGTGTGGGGTTTTG	−179 to −159
MSP-UR	CCAAACCAAATACATTTCTCCAA	−122 to −100
MSP-ML^d^	GGTGATTAGCGTGGGGTTTC	−179 to −160
MSP-MR	CGAACCAAATACGTTTCTCCG	−121 to −101

a L=(aggaagagag) + (gene-specific sequence), R=(cagtaatacgactcactatagggagaaggct) + (gene-specific sequence).

b MUC4-7L and MUC4-7R primers were designed for the antisense strand because PCR amplification was not obtained in the forward direction.

c U indicates the primer for unmethylated alleles.

d M indicates the primer for methylated alleles.
